# The thymus and the science of self

**DOI:** 10.1007/s00281-020-00831-y

**Published:** 2021-01-07

**Authors:** Vincent Geenen

**Affiliations:** GIGA Research Institute, GIGA-Immunity, Inflammation and Infection (GIGA-I3), University of Liège, Avenue de l’Hôpital CHU-B34, B-4000 Liège-Sart Tilman, Belgium

**Keywords:** Thymus, Self-peptides, Self-tolerance, Autoimmunity, Type 1 diabetes, Self-vaccination

## Abstract

The conventional perception asserts that immunology is the science of ‘discrimination’ between self and non-self. This concept is however no longer tenable as effector cells of the adaptive immune system are first conditioned to be tolerant to the body’s own antigens, collectively known as self until now. Only then attain these effectors the responsiveness to non-self. The acquisition of this essential state of tolerance to self occurs for T cells in the thymus, the last major organ of our body that revealed its intricate function in health and disease. The ‘thymus’ as an anatomical notion was first notably documented in Ancient Greece although our present understanding of the organ’s functions was only deciphered commencing in the 1960s. In the late 1980s, the thymus was identified as the site where clones of cells reactive to self, termed ‘forbidden’ thymocytes, are physically depleted as the result of a process now known as negative selection. The recognition of this mechanism further contributed to the belief that the central rationale of immunology as a science lies in the distinction between self and non-self. This review will discuss the evidence that the thymus serves as a unique lymphoid organ able to instruct T cells to recognize and be tolerant to harmless self before adopting the capacity to defend the body against potentially injurious non-self-antigens presented in the context of different challenges from infections to exposure to malignant cells. The emerging insight into the thymus’ cardinal functions now also provides an opportunity to exploit this knowledge to develop novel strategies that specifically prevent or even treat organ-specific autoimmune diseases.

## A short history of the thymus

The ancient Greek language uses two words that differ only by their accentuation: θυ^′^μος and θυμο^′^ς. The first one, accentuated on υ, is an ancient name of a plant, whereas the second, accentuated on ο, has been often used in philosophy to designate passion, soul, ardour and courage. As a medical term, thumos means ‘outgrowth’ in the pseudo-hippocratic text ‘*De alimento*’ and was also used in this meaning by Galenus (129 BC–ca. 210 AC) in his treaty ‘*De tumoribus praeter naturam*’. Under the generic term ‘ονκοσ’ (swelling), Galenus assembled different structural alterations including phlegmon, canker, polyp and others. Beyond the term’s generic use, Galenus specifically related the term to thymos, a gland situated behind the sternum. This specific concept was notionally present in discourse *‘About glands (peri adenon)’*, which is part of the Corpus Hippocraticum, a collection of early Ancient Greek medical works related to the physician Hippocrates and his teachings (5th century BC). In the understanding of Galenus, the retro-sternal position of the thymus served to protect the vena cava and other truncal vessels (comparable in today’s technical understand to an ‘airbag’). Nonetheless, Galenus also reported that the volume of the thymus demonstrated age-related changes as he noted during dissection a smaller organ in older monkeys (marmosets).

Significant advancement in our knowledge of thymus function was not made until the beginning of the twentieth century, because the scientific community believed that the thymus was just a vestigial and transitory organ, as extensively documented in a recent review devoted to the history of the thymus [1]. The Swedish physician Jan-August Hammar (1861–1946) was the first to report that dispersed fragments of normal thymus could be detected even in individuals of advanced age albeit the organ’s largest expansion was observed in puberty. Hammar also made several other important observations that have remained correct to our present days. For example, he noticed that the castration of animals before they reached puberty correlated with the persistence of a large thymus volume; that thymic involution was associated with pregnancy, undernourishment and several infectious diseases; and that thymus hyperplasia could be observed in patients with Graves’ hyperthyroidism, Addison’s adrenal insufficiency, myasthenia and acromegaly [2]. Following these original observations, the thymus was considered as an intrinsic glandular component of the endocrine system, and this idea was reinforced by studies of the pioneering Hungarian-Canadian endocrinologist Janos “Hans” Selye (1907–1982) who showed that thymic involution was promoted by stressful conditions [3]. However, it was impossible at that time to reconcile these observations with the presence of numerous lymphocytes within thymus (termed thymocytes) and the fact that thymectomy in fish and adult mice did not induce any diverse immune consequences of clinical significance.

The link between the thymus as an anatomical structure and its central importance for the function of the immune system was only established in the early 1960s of the last century when the French-born Australian scientist Jacques Francis Albert Pierre Miller observed that thymectomized newborn mice prematurely died secondary to a markedly increased susceptibility to infections and also failed to reject skin allografts, two essential hallmarks of immune functions used at that time to probe the system [4]. Given the extent of lymphopenia in blood, spleen and lymph nodes of mice thymectomized at birth, Miller logically concluded that the thymus must be responsible for this functional competence and thus referred the cells able to convey this immune function thymus-dependent lymphocytes, hence T cells. Despite Miller’s elegant experiments and concise conclusions, the British biologist Peter Medawar (1915–1987) still held in 1963 the understanding that ‘We shall come to regard the presence of lymphocytes in the thymus as an evolutionary accident of no very great significance’ [5]. In marked contrast to this incorrect statement by Medawar and as following up on his revolutionary theory on clonal selection, the Australian virologist and immunologist Frank Macfarlane Burnet (1899–1985) was already in 1962 at a University of London conference of the opinion: ‘If, as I think, the thymus is the site where occurs proliferation of lymphocytes in clones with precise immune functions, we have also to consider another function: elimination or inhibition of clones reactivity to Self’. This ultra-short historical summary highlights a few of the major milestones that eventually led to our current understanding of the role of the thymus in immunology. Needless to say, there are many seminal observations related to the history of the thymus that would also deserve to be mentioned here but had to be omitted due to space limitation for this review.

## Immune tolerance to self

In 1900, the German physician-scientist Paul Ehrlich (1854–1915) coined the dictum ‘horror autotoxicus’ to claim the impossibility that the human immune defence could be directed against its own cells. He therefore postulated the existence of mechanisms that prevent this form of autoimmunity and professed that these must be of the highest importance to individual’s life and for a species survival [6]. The notion that the immune system should not be reactive to self was later designated by Frank Macfarlane Burnet as ‘self-tolerance’, a term marking a fundamental property of the immune system that is closely related to immune diversity and specificity.

The molecular mechanisms accounting for T (TCR) and B (BCR) cell antigen receptor diversity, and hence an individual’s repertoire of antigen reactivity, were deciphered by Susumu Tonegawa in the 1970s and Mark Davis in the 1980s, respectively [7, 8]. A complex process of combinatorial DNA recombinations allows for a seemingly unrestricted number of individual antigen receptors (computed to be approximately 10^30^). Given the randomness by which antigen receptor specificities are generated during lymphocyte differentiation, it was however also realized that any initial repertoire of those receptor specificities would unquestionably include many reactivities against antigens of the host. These specificities would have, however, the potential to initiate and maintain autoimmunity. The immune system’s central purpose is the protection of benign self against injurious non-self. A concept had thus to be formulated that could explain how potentially harmful lymphocytes reactive to self are consequently prevented from further differentiation to the peripheral repertoire of antigen receptors. In the late 1980s, research groups led by Nicole Le Douarin in France, John Kappler, and Philippa Marrack in the USA and Hugh Robson Macdonald and Harald von Boehmer in Switzerland [9–12] recognized thymic stromal cells to be responsible for the negative selection of T cell clones reactive to self (termed ‘forbidden’ by Frank Macfarlane Burnet). As a consequence, negatively selected thymocytes are physically removed from the pool of immature lymphoid cells in the thymic microenvironment. Investigating the cellular events that control this selection also revealed that thymocytes were first probed to recognize the host’s major histocompatibility complex (MHC) molecules presenting peptides that originate from self-antigens. Only lymphocytes expressing a TCR with sufficient affinity for the self-peptide/MHC complex are able to survive at this stage in intrathymic maturation, whereas cells that express a TCR with insufficient affinity will undergo death by neglect. Hence, effective recognition of self is an important and unconditional prerequisite to progress to the thymic events that have functionally been defined as negative selection and that instruct tolerance to self-antigens. The resultant antigen receptor repertoire of thymocytes that have achieved positive and avoided negative selection defines the capacity of the pool of mature T cells to respond to non-self-antigens.

These seminal studies identified the thymus as an anatomical site where a relatively small number of T cells with tailored characteristics are selected from a large pool of immature thymocytes expressing collectively a seemingly unrestricted variance of antigen receptor specificities. In aggregate, the vast majority of T lymphocytes fail to survive their ontogenetic journey to reach a phenotypically mature stage as their TCR specificity is either incompatible to be positively selected or of a too high affinity for the recognition of self and consequently subject to negative selection. Of the very few blood-borne T cell precursors that enter the thymus and eventually enormously expanded to hundreds of millions of cells, only a very small fraction (estimated to less than 5%) will successfully complete their differentiation and exit the thymus as mature T cells tolerant to self yet reactive to non-self [13].

Negative clonal selection is not the only tolerogenic mechanism that establishes immune tolerance to self. Regulatory T cells (Tregs, reviewed elsewhere in this issue) provide an alternative, dominant mechanism to reinforce self-tolerance among peripheral T cells. Following an initial identification of lymphocytes with immunosuppressive capabilities by the American immunologist Richard Gershon (1932–1983) and initially termed suppressor T cells, it was the elegant work of the Japanese physician-scientist Shimon Sakaguchi and that of others which precisely identified the phenotype of Tregs and characterized their essential role in regulating the adaptive immune response [14]. Natural Tregs are generated in and exit from the thymus once the organ has reached morphological maturity that is early in the postnatal life of mice and prior to week 16 of human gestation. In reference to the anatomical site of their origin, these regulatory cells are also referred to as thymic (t) Tregs. This population functionally inhibits peripheral T cells with a TCR specificity highly reactive to self-antigens as they have escaped the rigours of thymic negative selection [15, 16].

In light of both recessive (negative thymocyte selection) and dominant (positive selection of tTreg cells) mechanisms that maintain immune self-tolerance, it may be provocative to speculate, as the American immunologist Polly Matzinger has previously conjectured [17], that there is no need for positive selection as this does not provide an evolutionary advantage over the establishment and use of a process of negative selection. Rather, the presentation of self-antigens in the context of MHC molecules on the surface of thymic epithelial cells (TECs) and other intrathymic antigen-presenting cells suffices to effect both negative selection and removal of forbidden T cell clones as well as positive selection of tTregs. Consequently, this line of thinking concludes that thymus output concerns only T cells that have escaped negative selection including those cells that have diverted to adopt a tTreg fate [18].

## The presentation of neuroendocrine self in the thymus

An international symposium held in 1983 entitled ‘Neural Modulation of Immunity’ [19] made reference to an observation already noted in 1910, namely, that the injection of extracts from the thymus could stimulate lactation in goats [20]. The galactagogue capacity of these extracts was later ascribed to oxytocin, a peptide neurohormone only later precisely characterized and eventually synthesized in the 1950s [21]. During axonal transport in hypothalamic magnocellular neurones, two precursor proteins are catalysed into oxytocin, vasopressin and their carrier proteins neurophysins, which are secreted from the posterior lobe of the hypophysis into the blood [22, 23]. In the thymus, oxytocin is produced in equimolar concentration with its neurophysin by a subset of thymus stromal cells known as TECs [24, 25]. Thymic nurse cells (TNCs) are a specific cellular site in the cortex of the thymus where oxytocin is produced [26]. TNCs are large cortical TECs that express β5t, a specific component of the thymoproteasome, which enclose many viable immature thymocytes that undergo secondary T cell receptor (TCR) α chain rearrangement to optimize T cell selection [27]. TNCs are an instructive example of cellular crosstalk enabling an intimate physical association between cells of distinct embryological origins, namely, a neuroendocrine-like and immune phenotypes. Interestingly, immature T cells express specific neurohypophysial receptors, and their binding of oxytocin phosphorylates downstream-placed proteins including effectors implicated in focal adhesion, thus possibly promoting the establishment of ‘immune synapses’ between TECs and thymocytes [28, 29]. These ‘synaptic’ structures between antigen-presenting cells and T lymphocytes are thought to play a fundamental role in intrathymic T cell differentiation and T cell activation in periphery [30].

Over time and with the advent of powerful molecular tools such as RNA sequencing, several other neuroendocrine-related transcripts could be identified that are expressed by broadly cortical and medullary TECs. Here, a hierarchical principle has been noted that relates to the repertoire of neuroendocrine effector molecules detected in these cells. For each functional family of proteins, a single, dominant member is expressed in the thymus. Namely, oxytocin represents the family of neurohypophysial hormones, neurokinin A that of tachykinins, cortistatin is the representative for the family of somatostatins, insulin-like growth factor 2 (IGF2) portrays the diverse group of insulins, the neuropeptide Y denotes the family of Y peptides, and neurotensin represents the neuromedin cluster [reviewed in 31, 32]. The transcription of loci encoding the neuroendocrine molecules in TECs had early been conceived to occur in a fashion distinct from the tightly regulated control operative in tissues where these molecules are characteristically synthesized and detected [33]. Though the precise molecular mechanism by which this promiscuous gene expression (PGE) is realized has in the last two decades been further elucidated [34], but several aspects of it remain incompletely understood. Most importantly, these peptide hormones are not synthesized and secreted to serve as classical neuroendocrine messengers but contribute a comprehensive collection of self-antigens (as exemplified here by the expression of several neuropeptides). These proteins are processed in TECs to form an array representing self, which are eventually presented in the context of MHC molecules to developing thymocytes. The recognition of the peptides/MHC complexes allows for the selection of TCR specificities and hence the generation of a repertoire of antigen specificities that has been educated on self to accomplish its functional utility. Moreover, there is experimental evidence that suggests that the thymic presentation of neuroendocrine self by thymic stromal cells is distinct from the constraints of non-self-antigen presentation by extrathymic antigen-presenting cells [as further discussed in references 35–38].

With mechanisms identified that enforce tolerance to self, a central question still remains unanswered, namely, how the repertoire of an individual’s own tissue-restricted antigens (TRAs) can be adequately represented within the thymus. This is, as outlined above, crucially important as peptides derived from those antigens are required to render the process of negative selection of effector T cells bespoke and the instruction of Treg cells possible. For considerable time, it had been assumed that antigens external to the thymus would be passively taken up from the circulation and captured in the thymus by dendritic cells (DCs) and macrophages that would act in this organ as professional antigen-presenting cells in this organ [39]. Though at first sight both conceivable and attractive, this concept nonetheless presumes that all (relevant) antigens are present in and accessible from circulation and that their thymic presentation suffices to establish T cell tolerance. However, we established in the late 1980s that genes encoding protein precursors to neuroendocrine hormones are transcribed and translated in the thymus, subsequently processed to peptides and eventually presented in the context with MHC. It is this intricate process by which the thymus exerts a specific and unique function establishing and maintaining central immune tolerance to the neuroendocrine system [40].

## Self-antigens and tissue-restricted antigens

The complexity in the thymic representation of tissue-restricted antigens (TRAs) was further appreciated when Doug Hanahan, an American biochemist, detected transcripts for pancreas-specific gene products to be expressed in very rare cells in the medulla [41, 42]. Subsequent research led by the late German immunologist Bruno Kyewski further extended the notion of PGE to apply a large number of loci that encode TRAs. His and the work of others provided thus solid experimental evidence that the non-canonical transcription of genes typically expressed in a restricted fashion in tissues other than the thymus was key to sanction central immune tolerance to self and thus essential to prevent the risk of autoimmunity [43, 44]. Notably and in contrast to the aforementioned expression of neuroendocrine proteins which are expressed in all TEC subpopulations, genes encoding TRAs are typically transcribed only in a subset of medullary (m) TECs in a transient fashion at lower levels than typically observed in peripheral organs, and, occasionally, in patterns that correspond to their position along chromosomal clusters [45]. In addition to their role as intrathymic APCs controlling the thymic selection of the TCR repertoire, mTECs have also been identified to release TRAs that can be taken up for antigen presentation by thymus resident DCs, thus broadening the number and type of cells that probe TCR specificities within the thymic medulla [46]. Following an initial scepticism, the crucial importance of thymus-dependent, i.e. central tolerance enforced by the representation of TRAs, was broadly recognized and forms today an integral part of our understanding of the functional importance of the thymus in shaping the reactivity of the adaptive immune system [31, 47, 48].

In light of the molecular insight gained in the course of two decades of thymus research, it emerges that there are two parallel, yet complementary mechanisms that secure an efficient and close to comprehensive establishment of central tolerance. One mechanism concerns the way how neuroendocrine self-peptides are made available throughout the thymus, with IGF2 and oxytocin being typical exemplars that translate into a robust process of tolerance induction encompassing most of the molecular families of the neuroendocrine system. The other mechanism is characterized by PGE and relates to genes encoding TRAs (such as insulin and vasopressin), which are expressed at any given time only by a subset of specialized TECs in the medulla. This second mechanism therefore appears to be less potent with regard to the stringency how antigen-specific tolerance is imposed. The ensuing differences in self-representation are important as they establish an apparent hierarchy in effectiveness relevant for evolution.

The necessity to be tolerant to neuroendocrine self will have emerged during phylogeny and parallel to an increased complexity of antigens arisen from further cell differentiation and specialization. This mechanism of tolerance would have needed to be successfully in place before an adaptive immune system had developed. Related to the example given above, oxytocin is essential for different steps in reproduction and is thus essential for the preservation of species that produce this hormone and depend on it. In contrast, the expression of the neurohypophysial peptide vasopressin, which primarily regulates water homeostasis and vascular tone, appears to be less conducive to establish effective antigen tolerance. Antibodies against vasopressin have been identified in patients with idiopathic autoimmune central diabetes insipidus (and other diseases like Langerhans cell histiocytosis and germinomas), while autoimmunity targeting hypothalamic oxytocinergic neurons has not been observed to date. Noteworthy, the recognition by a specific monoclonal antibody of thymic oxytocin expressed at the membrane of TECs induced production by TECs of cytokines helpful for development and survival of thymocytes not directed to oxytocin [36]. Similarly, autoimmunity caused by recognition of IGF2, a factor essential for foetal growth and development, has also not been reported whereas insulin serves as a primary autoantigen in the context of type 1 diabetes (T1D).

These experimental observations lend themselves to speculate that there may be a distinction necessary between tolerogenic self-antigens and immunogenic autoantigens. The English language uses the terms self-antigen and autoantigen interchangeably though the terms ‘self’ and ‘auto’ have different etymological origins and thus introducing a possible semantic dimension. The Latin word for *self* (‘sei’) refers to something that is reflexive to the person, while the Greek word *auto* is used to signify spontaneity. With regard to the need to differentiate between these two, conceptually distinct entities of antigens, IGF2 would need to be referred as the *self*-antigen of the whole insulin family, while (pro)insulin per se is to be classified as the primary *auto*antigen in T1D. Supporting this concept, tolerance to insulin is markedly decreased in *Igf2*^-/-^ mice, which suggests that expression of IGF2, as self-peptide of the insulin family, is necessary for the establishment of a complete immune tolerance to insulin [49]. The small biochemical difference between IGF2 and insulin could drive two opposite outcomes of adaptive immune response, tolerogenicity vs. immunogenicity.

## Autoimmunity as a failure of thymus-dependent self-tolerance

Macfarlane Burnet used the term ‘forbidden’ to characterize T cell clones with a reactivity to self and predicted as early as 1973 that these cells could play a major role in events leading to autoimmune pathologies [50]. Several occasional observations tried to associate a thymus dysfunction with pathological events culminating in the manifestation of T1D (reviewed in reference 51). Hence, it was deliberated whether a fundamental defect in the MHC-mediated presentation of self-antigens and/or TRAs during TCR selection could result in a steady output of ‘forbidden’ effector T cells and/or tTreg cells of a disease-relevant antigen specificity. If this supposition were to be confirmed, a thymus dysfunction would constitute the earliest event in the generation of antigen-specific T cells promoting organ-specific autoimmunity.

The defect in *Igf2* expression in the thymus of a specific strain of diabetes-prone Bio-Breeding rats (DP-BB), an animal model of T1D [52], and the low level of *Ins2* transcripts in the thymus of human fetuses with genetic susceptibility to T1D were therefore among the first experimentally proven evidenced supporting the above hypothesis [53, 54]. Thymic T cell tolerance induction is also influenced by *Ins2* expression [55], which in turn is modified by the insulin-specific transactivator Mafa. Polymorphisms of the gene encoding this factor, *Mafa*, result in reduced insulin expression in TECs and are associated with a heightened susceptibility both in a mouse model and in human for T1D [56].

Transcripts for the complete array of thyroid-specific antigens are under normal conditions present in human TECs [57–59]. Moreover, individuals homozygous for an SNP predisposing to Graves’ autoimmune thyroiditis have been documented to express lower intrathymic transcripts of *TSHR* [60]. Comparable findings have also been noted for non-endocrine organ systems. For example, the physiological lack of α-myosin expression in TECs precludes the induction of T-cell tolerance to this cardiac antigen and enables T cell priming in the context of a heart attack complicating the vascular consequences with the development of autoimmune myocarditis [61]. Other examples concern a number of neurological pathologies where experimental evidence and clinical observations would strongly argue for a central role of thymus-dependent tolerance in the pathogenesis of these disorders [62].

The autoimmune polyendocrinopathy syndrome 1 (APS-1) provides to date the most convincing example that thymus function constitutes an essential primary defence line against organ-specific autoimmunity as it prevents inadequate TCR repertoire selection to an absence of appropriate self-representation by TECs [63]. The molecular cause for this debilitating pathology affecting endocrine and non-endocrine organs is an autosomal recessive deficiency in the expression of the autoimmune regulator (AIRE), a member of the family of PhD-domain containing zinc fingers [64, 65]. Aire expression is limited to a subset of phenotypically mature mTECs that coexpress also several ligands for the complete signal transduction upon TCR engagement by thymocytes [66]. Aire expression is regulated in the thymus by members of the tumour necrosis factor receptor superfamily such as Tnfrsf1a (also known a receptor activator of nuclear factor-κB, RANK) and CD40 [67]. An extrathymic expression of AIRE has also been observed in mice in rare subset of bone marrow–derived cells and shown to induce functional inactivation of peripheral CD4+ T cells [68]. In mice, the transplantation of an *Aire*^-/-^ thymic stromal compartment induces organ-specific autoimmunity paralleled by the decrease in the intrathymic transcription of genes encoding neuroendocrine self-antigens (oxytocin, IGF2) and many TRAs [63]. The action of AIRE depends on several epigenetic constraints such as repressive post-translational histone modifications and special chromatin configuration [69]. In mice, only the expression of approximately 4000 TRA-encoding genes are, however, completely or partially dependent on AIRE expression [70]. Hence, the search for other factors that enable PGE has been at the centre of research of several groups. A putative second mechanism enabling PGE in TECs seemingly employs the neuronal transcription factor Fezf2, which recognizes a number of TRA-encoding loci different from those controlled by AIRE [71]. Interestingly, both AIRE and Fezf2 depend for the transcription of some but not all of their target genes on the ubiquitously expressed chromatin remodelling enzyme Chd4 [72]. This common dependence on a molecule that recognizes repressive histone marks suggests that both AIRE and Fezf2 take advantage of comparable molecular mechanisms for their activity. Fezf2 has originally been identified to be required for brain differentiation and specification [73] and thus also serves purpose outside of the thymus, similar to AIRE. Notably, neither *Aire* nor *Fezf2* transcription is changed with age (at least in mouse) but PGE of AIRE-controlled genes is diminished at later ages, suggesting a mechanism of transcription that is also reliant on factors other than AIRE abundance [74]. This mechanism still awaits to be precisely defined.

## The concept of tolerogenic *inverse* self-vaccination against T1D

The immune response observed in T1D leading to the immune destruction of insulin-secreting islet β cells with its clinical consequences primarily results from an immunogenic response to the disease’s main autoantigen, insulin. The lack of tolerance to insulin and the subsequent reactivity to this antigen may arise, as mentioned above, from an infrequent, transient and low expression of *INS2*/*Ins2* by single cells of a restricted subset of mTECs. Restoring immune tolerance to islet β cells holds therefore the potential to halt the destructions in patients already diagnosed to have T1D or to prevent the cells’ attack in individuals with high risk for T1D. One clinical study inferred that the nasal exposure of adults with recent-onset T1D to specific tolerance. This treatment did not prevent the destruction of their islet β cells [75]. Another study treated adult patients diagnosed with T1D within 5 years with proinsulin [76]. Though a reduced frequency of CD8+ T cells against insulin but not unrelated islet or foreign antigens and preservation of C-peptide levels were observed over the course of the study, it remains unknown whether this treatment and other clinical trials based on insulin tolerization influenced the course of T1D and protected the residual β cells mass from the diabetogenic autoimmune response over time. A novel type of vaccine strategy, operationally termed ‘inverse self-vaccination’, hence takes advantage of the fact that the self-antigen IGF2 is under experimental conditions highly tolerogenic [77]. The rationale of employing IGF2 to tolerize against insulin is built on several experimental observations and takes into account that proinsulin does not have any tolerogenic properties that could be used to reprogram immune tolerance toward islet β cells as mentioned above. For example, *Igf2* is the dominant member of the insulin gene family that is expressed in TECs [78] and its deletion coincides with a marked decrease in tolerance to insulin, thus suggesting a significant potency for IGF2 to cross-tolerize [49]. Persistent infection of mTECs with the ‘diabetogenic’ coxsackievirus B4-E2 results in vitro in a decreased production of IGF2 [79]. In vivo, *Igf2* transcription is also detected in the thymus of diabetes-resistant BB rats characteristically resistant in the spontaneous development of autoimmune diabetes. However, *Igf2* transcripts and IGF2 protein are absent in the thymic microenvironment of a large majority (around 85%) of diabetes-prone BB rats in close correspondence with the rate of diabetes incidence in this BB strain [52]. Moreover, peptides derived from IGF2 (B11-25) and insulin B9-23 compete for binding to two MHC class II haplotypes (DQ2 and DQ8), which are known to confer the highest genetic susceptibility to T1D (unpublished data). Extending on this observation, mononuclear cells isolated from DQ8+ diabetic adolescents that present in vitro IGF2 B11-25 but insulin B9-23 induce a tolerogenic cytokine profile marked by the induction of IL-10 expression [80]. Complementary to these findings, IGF2 has been noted to activate regulatory B cells directed against primed, heterologous antigen [81], as well as Treg cells competent to suppress other effector T cells driving an inflammatory immune response [82].

## Conclusion (Fig. 1)

Global co-evolution of the neuroendocrine and the immune systems also deserve some attention. Since their appearance, the neuroendocrine and innate immune systems have evolved and still coexist nowadays without any apparent problem. This harmony may result from the fact that the principal mediators of innate immune cells, Toll-like receptors (TLRs), do not seem, at least until now, to have the ability to react against normal or undamaged self. Primitive forms of anticipatory immunity exist in agnathans and are mediated by 4–12 leucine-rich repeat modules (VLR) most probably assembled by gene conversion [83]. Around 450 million years ago, the appearance of transposon-like recombinase-activating genes (*Rag1* and *Rag2*) enabled the generation of a seemingly unrestricted diversity of antigen receptors via a process of stochastic recombinations which was termed the ‘Big Bang’ of the immune system by some authors [84]. As discussed above, this remarkable generation of diversity is inherently associated with a high risk of autotoxicity/autoimmunity. Hence, as evolution progressed, the sparse thymus-like lympho-epithelial structures, termed thymoids, in the branchial apparatus of agnathans [85], needed to give way in sharks and rays to a primary lymphoid organ better adapted to quality control a randomly selected repertoire of TCR specificities. In parallel, the capacity to transcribe and present dominant TRAs including neuroendocrine self-peptides was required as an essential mechanism to subject immature thymocytes to a rigorous education that assures immunological tolerance to peripheral tissues.

**Fig. 1:**
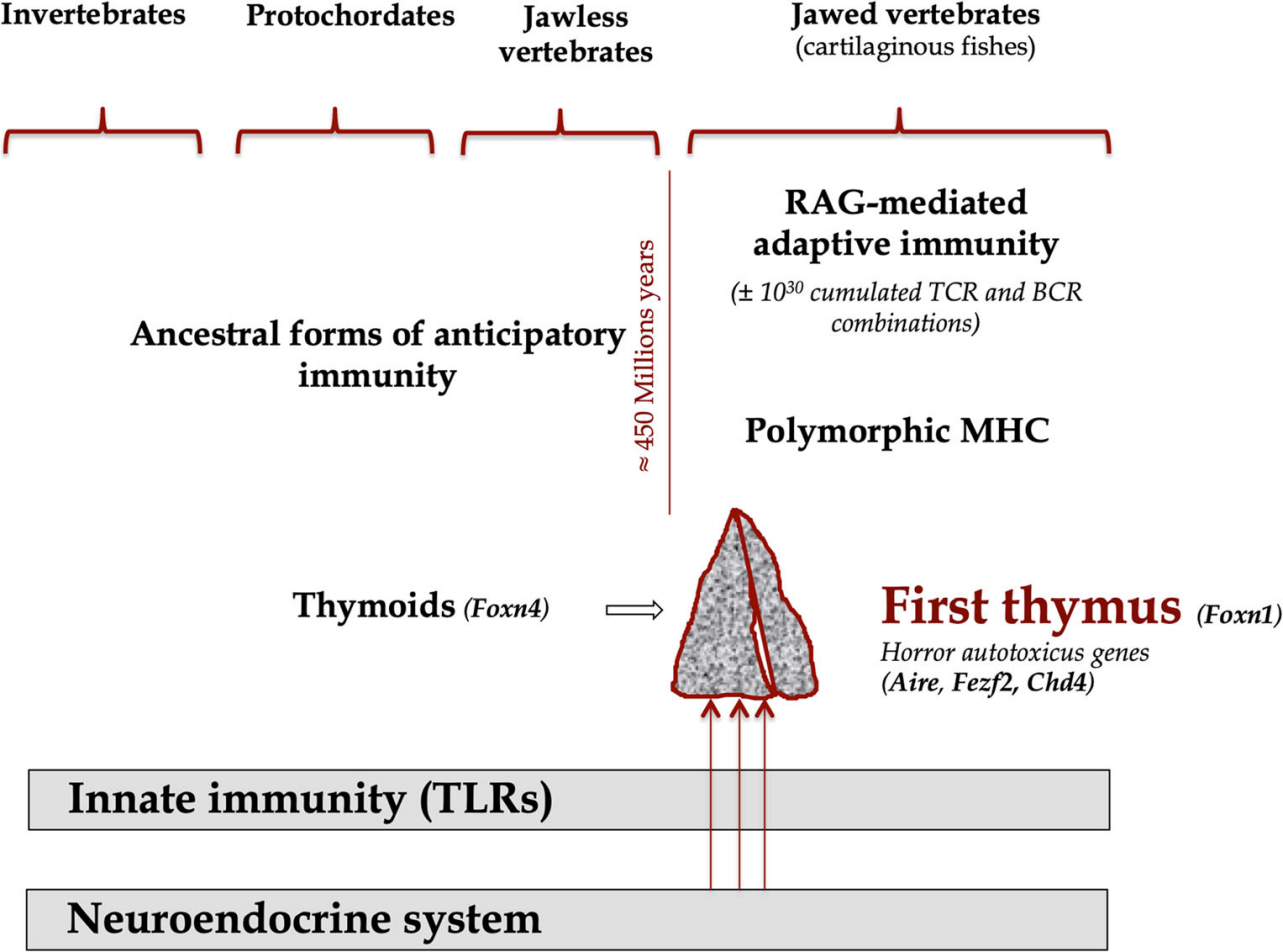
Integrated and harmonious co-evolution of the neuroendocrine and immune systems**.** Please see a full description of this figure in the ‘Conclusion’ section of this review

Immunological self-tolerance to the neuroendocrine system had to evolve as a necessity since many hormones and neuropeptides shape the immune response via binding to and activation of their respective receptors on effector cells of the immune system. In the absence of self-tolerance to these ligands and receptors, the risk of developing neuroendocrine autoimmunity would be debilitatingly high and with it the health of an individual in jeopardy and the existence of a species in peril.
